# Further suggestions on the group-theoretical approach using clinical values

**DOI:** 10.1186/1742-4682-9-54

**Published:** 2012-12-19

**Authors:** Jitsuki Sawamura, Shigeru Morishita, Jun Ishigooka

**Affiliations:** 1 Department of Psychiatry, Tokyo Women’s Medical University, Tokyo, Japan; 2 Depression Prevention Medical Center, Kyoto Jujo Rehabilitation Hospital, Kyoto, Japan

**Keywords:** Group theory, Modulo operation, Prescription, Laboratory data, TNM classification, ICD coding schemes

## Abstract

**Background:**

In a previous report, we suggested a prototypal model to describe patient states in a graded vector-like format based on the modulo groups via the psychiatric rating scale. In this article, using other simple examples, we provide additional suggestions to clarify how other clinical data can be treated practically in line with our proposed model.

**Methods:**

As illustrations of the wider applicability, we treat four cases commensurate with modulo arithmetic: 1) prescription doses of three medicines (lithium carbonate, mirtazapine, and nitrazepam), 2) changes in laboratory data (blood concentrations of lithium carbonate, white blood cells, percutaneous oxygen saturation and systolic blood pressure), 3) the tumor node metastasis (TNM) classification of malignant tumors applied for esophageal tumors, and 4) the coding schemes of the International Classification of Diseases (ICD) for selected diseases or laboratory data. For each case, we present simple examples in the form of product of states to illustrate these results.

**Results:**

1) Medications and their changes can be represented as elements of a modulo group; e.g., group S = {S_j_ | S_j_ ∈ Z_13_×Z_4_×Z_3_} can represent the set of all possible prescription combinations of three specified medicines. Likewise, 2) clinical values can also be expressed as a modulo group; e.g., group T = {T_j_ | T_j_ ∈ Z_600_×Z_50000_×Z_100_×Z_300_} representing the set of all possible data based on any number of clinical values and their differences. Also, 3) the TNM classification for malignant tumors can be treated within a single modulo group C = {C_j_ | C_j_ ∈ Z_8_×Z_4_×Z_2_×Z_2_}, the set of all composable disease states graded in terms of tumor expansion. Finally, 4) ICD coding schemes provide several examples treatable as a modulo group D = {D_j_ | D_j_ ∈ Z_7_×Z_7_× …×Z_7_ (an n-fold product)}, constituting the set of all possible severities of diseases states and laboratory data within provided tuples.

**Conclusions:**

Despite the limited scope of our methodology, there are grounds where other clinical quantities (prescription of medicine, laboratory data, TNM classification of malignant tumors, and ICD coding schemes) can be also treatable with the same group-theory approach as was suggested for psychiatric disease states in our previous report.

## Background

Group theory is one of the cornerstones in various branches of natural science, providing enormous advantages for their respective fields [1-4]. Unfortunately, medicine has not been sufficiently systematized in general, and has not attained a level of sophistication linking it directly with other fields of natural science. One reason for that is the lack in medical science of a group-theory systematics, in particular, an effective notational style. Considering these aspects, we have previously suggested a prototypal model with the potential to describe patient states in a graded vector-like (or Cartesian product) notation based on the modulo group via the psychiatric rating scale [5].

Briefly, in our previous report, using the BPRS-I (the virtually modified original Brief Psychiatric Rating Scale that is frequently used for the estimation of psychiatric disease states), we demonstrated the group-theoretical relationship in the style:

$$‘A_{j}*A_{\left(j\to k\right)}=A_{j}\left(\text{mod}\;7\right)+A_{\left(j\to k\right)}\left(\text{mod}\;7\right)=\left\{A_{j}+A_{\left(j\to k\right)}\right\}\;\left(\text{mod}\;7\right)=A_{k}\left(\text{mod}\;7\right)’$$

where j,k = 1,2,3,…; (positive integers), A_j_ denotes a patient’s disease state expressing a combination of the respective symptom severities, and A_(j→k)_ is an operator that changes the disease state to A_k_ by acting, in the group-theory sense, on A_j_. Here, the composition of the operator A_(j→k)_ with the state A_j_ follows the ‘right translation’ rule, that is, operators act from the right side of the state.

We presume that this method is also applicable in principle to other psychiatric evaluation scales such as the ‘Positive and Negative Syndrome Scale’ (PANSS) for schizophrenia [6], the ‘Montgomery Åsberg Depression Rating Scale (MADRS)’ for depression [7], and almost all psychiatric rating scales so long as an appropriate number of modulo operations is chosen (as explained in our previous report, prime numbers are preferred).

In that report, A_j_, A_k_, and A_(j→k)_ are elements belonging to the same group (Z_7_^×18^, *) and all possible assessments within the provided psychiatric rating scale; note that to simplify the discussion, the scoring range, ‘1–7’, of each symptom was modified to ‘0–6’ to treat elements within a single group based on modulo addition (if not modulo multiplication and division). A patient’s state is changed only under the operations between the elements within that group. Not having to use modular operations connecting states of different patients is one of the advantages of the proposed model; the magnitude of data and/or handling requirements of patient medical records is considerably reduced.

The focus in the report was restricted in the main to demonstrate handling of psychiatric disease states as an example. Only fragmentary suggestions were given for use with, for example, laboratory data results, the tumor node metastasis (TNM) classification of malignant tumors [8], and the International Statistical Classification of Diseases and Related Health Problems, 10th Revision (ICD-10) [9]. Various aspects were not investigated sufficiently. In this article, we make redress by providing additional clarification on how in practice other clinical data can be treated in line with our proposed model using other simple examples.

### Further demonstrations of the model using clinical values

#### Applications to prescription dosages and other clinical data

To demonstrate applicability of the proposed model, we use medicine prescription levels. We assume the following scenario; when we treat a patient with bipolar disorder, for instance, we prescribe lithium carbonate, mirtazapine, and nitrazepam (see Table 1). We suppose the doses for each can be expressed in a vector-like form ‘S_j_’ that contains the dose of lithium in the 1^st^ component, that of mirtazapine in the 2^nd^ component, and that of nitrazepam in the 3^rd^ component; the vector S_j_ is denoted S_1_ = [lithium carbonate (mg/d)| mirtazapine (mg/d)| nitrazepam (mg/d)]. For example, S_1_ of the first session can be described as: S_1_ = [0 (mg/d)| 0 (mg/d)| 0 (mg/d)]. In the same manner, we can consider for the 2^nd^, 3^rd^, 4^th^, and 5^th^ sessions, prescription vectors:

$$\begin{array}{l} S_{2}=\left[200\;\left(\mathrm{mg}/d\right)\left|\;15\;\left(\mathrm{mg}/d\right)\right|\;5\;\left(\mathrm{mg}/d\right)\right],\\ S_{3}=\left[400\;\left(\mathrm{mg}/d\right)\left|\;30\;\left(\mathrm{mg}/d\right)\right|\;10\;\left(\mathrm{mg}/d\right)\right],\\ S_{4}=\left[600\;\left(\mathrm{mg}/d\right)\left|\;45\;\left(\mathrm{mg}/d\right)\right|\;5\;\left(\mathrm{mg}/d\right)\right],\\ S_{5}=\left[300\;\left(\mathrm{mg}/d\right)\left|\;30\;\left(\mathrm{mg}/d\right)\right|\;0\;\left(\mathrm{mg}/d\right)\right]. \end{array}$$

**Table 1:** An example of prescription dosages for a patient with bipolar disorder

	**Session 1**	**Session 2**	**Session 3**	**Session 4**	**Session 5**
Lithium carbonate	0 mg/d	200 mg/d	400 mg/d	600 mg/d	300 mg/d
Mirtazapine	0 mg/d	15 mg/d	30 mg/d	45 mg/d	30 mg/d
Nitrazepam	0 mg/d	5 mg/d	10 mg/d	5 mg/d	0 mg/d

Lithium carbonate, mirtazapine, and nitrazepam are prescribed and doses adjusted.

Next, we introduce modulo addition so that the S_j_ (j = 1,2,…,5) have the respective optimized numbers under modulo addition according to the individual restriction on each component. If supplemented with the modulo divisor ‘x_i_’ (the number that codes the modulo operation in i-th component of S_j_, i= 1,2,3), then ‘x_i_’ must be that number for which ‘x_i_ -1’ multiplied by the unit dose (e.g., ‘100 (mg/d)’ lithium carbonate) approximates the maximum of its clinical dosage. E.g., the lithium carbonate dosage would be given over thirteen sessions ‘0 = 0{100}, 100 = 1{100}, 200 = 2{100}, 300 = 3{100},…, 1200 = 12{100}’; thus, the maximum lithium carbonate dosage corresponds to ‘1200 = 100·(x_1_ - 1)’ giving ‘x_1_ = 13’, Hence, modulo 13 addition is the operation defined for the 1^st^ component (lithium carbonate). Likewise for mirtazapine; given the maximum dose of 45 (mg/d), we find ‘45 = 15·(x_2_ - 1)’ yields ‘x_2_ = 4’, and thus determines modulo 4 addition for the 2^nd^ component. Similarly, with the maximum of nitrazepam of 10 mg/d, ‘10 = 5·(x_3_ - 1)’ gives ‘x_3_ = 3’, and hence, modulo 3 addition for the 3^rd^ component. We can now rewrite the prescription vectors ‘S_j_’s in the form:

$$\begin{array}{l} S_{1}=\left[100\left\{0\;\left(\mathrm{mod}\;13\right)\right\}\left|\;15\left\{0\;\left(\mathrm{mod}\;4\right)\right\}\right|\;5\left\{0\;\left(\mathrm{mod}\;3\right)\right\}\right]\;\left(\mathrm{mg}/d\right)\\ S_{2}=\left[100\left\{2\;\left(\mathrm{mod}\;13\right)\right\}\left|\;15\left\{1\;\left(\mathrm{mod}\;4\right)\right\}\right|\;5\left\{1\;\left(\mathrm{mod}\;3\right)\right\}\right]\;\left(\mathrm{mg}/d\right)\\ S_{3}=\left[100\left\{4\;\left(\mathrm{mod}\;13\right)\right\}\left|\;15\left\{2\;\left(\mathrm{mod}\;4\right)\right\}\right|\;5\left\{2\;\left(\mathrm{mod}\;3\right)\right\}\right]\;\left(\mathrm{mg}/d\right)\\ S_{4}=\left[100\left\{6\;\left(\mathrm{mod}\;13\right)\right\}\left|\;15\left\{3\;\left(\mathrm{mod}\;4\right)\right\}\right|\;5\left\{1\;\left(\mathrm{mod}\;3\right)\right\}\right]\;\left(\mathrm{mg}/d\right)\\ S_{5}=\left[100\left\{3\;\left(\mathrm{mod}\;13\right)\right\}\left|\;15\left\{2\;\left(\mathrm{mod}\;4\right)\right\}\right|\;5\left\{0\;\left(\mathrm{mod}\;3\right)\right\}\right]\;\left(\mathrm{mg}/d\right) \end{array}$$

In this regard, these are Cartesian vectors accompanied with the dose unit ‘mg/d’; the integers ‘x_i_’ following ‘mod’ are the divisors of the modulo operation (s mod x), which yields the remainder after dividing s by x. The general form of the prescription vectors is as follows:

$$S_{j}=\left[100\left\{{\text{s}}_{\left(\text{j}\right)1}\left(\mathrm{mod}\;13\right)\right\}\left|\;15\left\{{\text{s}}_{\left(\text{j}\right)2}\left(\mathrm{mod}\;4\right)\right\}\right|\;5\left\{{\text{s}}_{\left(\text{j}\right)3}\left(\mathrm{mod}\;3\right)\right\}\right]\;\left(\mathrm{mg}/\text{d}\right)$$

where s_(j)1_, s_(j)2_, s_(j)3_ are positive integers and j = 1,2,…,n with n the total number of sessions for patient observation. The (mg/d) following vector is the common unit for all the components s_(j)i_ in S_j_; it is also permissible to have this unit included with the individual components as presented previously and exemplified in another example below.

**Figure 1:**
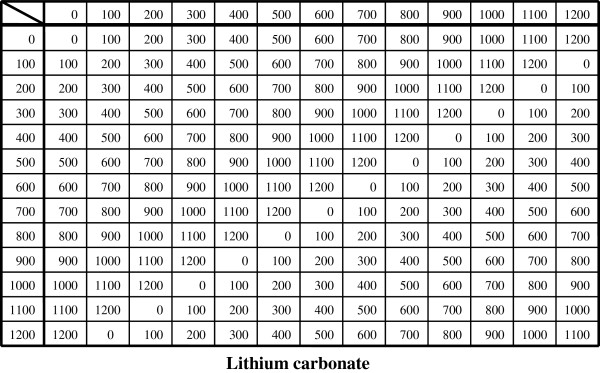
**Cayley table for dosages (mg/d) of ‘lithium carbonate’.** When divided by 100 (the unit dose of ‘lithium carbonate’), the numbers represent elements of C_13_={0,1,2,3,…,11,12} with modulo 13 addition.

**Figure 2:**
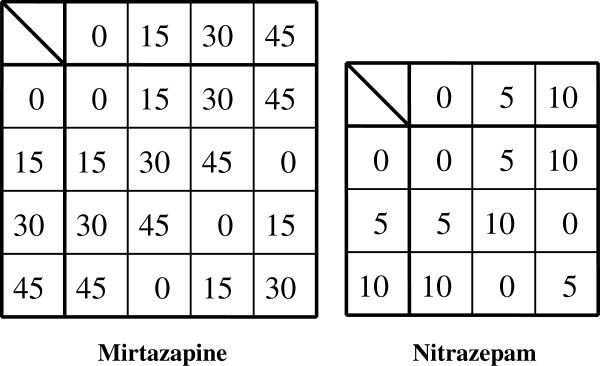
**Cayley tables for dosages (mg/d) of ‘mirtazapine’ and ‘nitrazepam’.** For the left table, numbers represent elements of C_4_={0,1,2,3} with modulo 4 addition when divided by 15 (the unit dose of ‘mirtazapine’). Similarly, for the right table, when divided by 5 (the unit dose of ‘nitrazepam’) the numbers represent elements of C_3_={0,1,2} with modulo 3 addition.

We can confirm the ‘S_j_’s also obey the group postulates because there is an identity element ‘0’, and an inverse element of ‘s_(j)i_’ denoted ‘s_(j)i_^-1^’ of the form ‘s_(j)i_^-1^= x_i_ - s_(j)i_’ for all components (‘x_i_’ being specifically x_1_= 13, x_2_= 4, x_3_= 3). By regarding ‘E = [100{0 (mod 13)}| 15{0 (mod 4)}| 5{0 (mod 3)}] (mg/d)’ as an identity element (no prescription) and ‘S_j_^-1^= [100{s_(j)1_^-1^ (mod 13)}| 15{s_(j)2_^-1^ (mod 4)}| 5{s_(j)3_^-1^ (mod 3)}] (mg/d) = [100{x_1_ - s_(j)1_ (mod 13)}| 15{x_2_ - s_(j)2_ (mod 4)}| 5{x_3_ - s_(j)3_ (mod 3)}] (mg/d)’ as the inverse element of S_j_, we can compose group S = {S_j_ (j = 1,…,W_S_)| S_j_ ∈ Z_13_×Z_4_×Z_3_}, the set of all possible prescriptions consisting of the three specified drugs. In this regard, |S| (the order of group S) is |S|≡13×4×3, The group composition law, denoted by ‘*’, is modulo addition for each modulo group Z_m_, the set {0, 1, 2,…, m - 1} with ‘m’ a positive integer (Cayley tables are shown in Figures 1, 2). Note that group S contains the non-prime modulo group Z_4_. Because inverses are non-unique for modulo groups based on non-primes, such Z_m_ cannot be develop into algebraic structures called rings or fields. In this respect, S is limited to just modulo addition (and subtraction). In such instances, there might be a loss in potential applicability in a more general systematization available with matrices. 

Following our previous report, we construct a transition operator S_(j→k)_ that, when given state S_j_, outputs state S_k_ by acting on state S_j_. This is defined as S_(j→k)_ ≡ S_j_^-1^*S_k_ =S_k_ - S_j_ and is seen as a difference between the two states S_j_ and S_k_. For various elements S_j_ of S, we present explicit expressions for possible transition operators (see Appendix A for details).

$$\begin{array}{l} S_{\left(1\to 2\right)}=\left[100\left\{2\;\left(\mathrm{mod}\;13\right)\right\}\left|\;15\left\{1\;\left(\mathrm{mod}\;4\right)\right\}\right|\;5\left\{1\;\left(\mathrm{mod}\;3\right)\right\}\right]\;\left(\mathrm{mg}/d\right)\\ S_{\left(2\to 3\right)}=\left[100\left\{2\;\left(\mathrm{mod}\;13\right)\right\}\left|\;15\left\{1\;\left(\mathrm{mod}\;4\right)\right\}\right|\;5\left\{1\;\left(\mathrm{mod}\;3\right)\right\}\right]\;\left(\mathrm{mg}/d\right)\\ S_{\left(3\to 4\right)}=\left[100\left\{2\;\left(\mathrm{mod}\;13\right)\right\}\left|\;15\left\{1\;\left(\mathrm{mod}\;4\right)\right\}\right|\;5\left\{2\;\left(\mathrm{mod}\;3\right)\right\}\right]\;\left(\mathrm{mg}/d\right)\\ S_{\left(4\to 5\right)}=\left[100\left\{10\;\left(\mathrm{mod}\;13\right)\right\}\left|\;15\left\{3\;\left(\mathrm{mod}\;4\right)\right\}\right|\;5\left\{2\;\left(\mathrm{mod}\;3\right)\right\}\right]\;\left(\mathrm{mg}/d\right) \end{array}$$

Naturally, we can easily confirm subsequent relationships among each prescription states (see Appendix B for details). Thus, we can verify the transition

$$\begin{array}{l} {\text{S}}_{1}*{\text{S}}_{\left(1\to 2\right)}*{\text{S}}_{\left(2\to 3\right)}*{\text{S}}_{\left(3\to 4\right)}*{\text{S}}_{\left(4\to 5\right)}\\ ={\;\text{S}}_{1}+{\;\text{S}}_{\left(1\to 2\right)}+{\;\text{S}}_{\left(2\to 3\right)}+{\;\text{S}}_{\left(3\to 4\right)}+{\;\text{S}}_{\left(4\to 5\right)}\\ =\left[100\left\{3\;\left(\mathrm{mod}\;13\right)\right\}\left|\;15\left\{2\;\left(\mathrm{mod}\;4\right)\right\}\right|\;5\left\{0\;\left(\mathrm{mod}\;3\right)\right\}\right]\;\left(\mathrm{mg}/\text{d}\right)\\ =\left[300\;\left(\mathrm{mg}/\text{d}\right)\left|\;30\;\left(\mathrm{mg}/\text{d}\right)\right|\;0\;\left(\mathrm{mg}/\text{d}\right)\right]\\ ={\;\text{S}}_{5}. \end{array}$$

Note, operator compositions follow the ‘right translation’ rule, that is the operator acts from the right side of the state, S_j_*S_(j→k)_ = S_k_. The preceding results indicate that drug prescriptions might also be amenable as group-theoretical operations within a single group S comprising all possible prescription combinations of three distinct but specified drugs.

In this way, not only multiple medical prescriptions, but other clinical data can also be treated in a similar style. Although regarding patient states as vectors may be peculiar from a meaningful pathological perspective, we believe that the way to use the proposed model might indicate further potential approaches to handling various clinical results. Specifically, we consider transitions in blood concentration of lithium carbonate ([Li^+^]), white blood cell (WBC), percutaneous oxygen saturation (SpO_2_), and systolic blood pressure (SBP), as listed in Table 2. The only necessary condition is that these results are non-negative real numbers with the inclusion of ‘0’. The order of each modulo group needs to be pre-defined so that it corresponds to maximum values. The vectors of the state presented in Table 2 can be expressed using the appropriate modulo additions. The order of the clinical values is as follows:

$$\begin{array}{l} T\;=[\left[{\mathrm{Li}}^{+}\right](\mathrm{mEq}/l)|\mathrm{WBC}\left(/{\mathrm{mm}}^{3}\right)|{\mathrm{SpO}}_{2}(\%)|\mathrm{SBP}(\mathrm{mmHg})]\;\mathrm{and}\\ T_{1}=[0(\mathrm{mEq}/l)|\;4000\;(/{\mathrm{mm}}^{3})|98(\%)|120(\mathrm{mmHg})]. \end{array}$$

**Table 2:** An example of a transition of various clinical values

	**Session 1**	**Session 2**	**Session 3**	**Session 4**
[Li^+^]	0 mEq/l	0.10 mEq/l	0.15 mEq/l	0.51 mEq/l
WBC	4000/mm^3^	5400/mm^3^	12000/mm^3^	6500/mm^3^
SpO_2_	98%	82%	93%	99%
SBP	120 mmHg	145 mmHg	105 mmHg	95 mmHg

Abbreviations: [Li^+^], blood concentration of lithium carbonate; WBC, white blood cell; SpO_2_, percutaneous oxygen saturation, and SBP: systolic blood pressure.

Similarly for the 2^nd^, 3^rd^, and 4^th^ sessions:

$$\begin{array}{l} T_{2}=[0.10\;\left(\mathrm{mEq}/l\right)|\;5400\;(/{\mathrm{mm}}^{3})|\;82(\%)|\;145\;(\mathrm{mmHg})],\\ T_{3}=[0.15\;\left(\mathrm{mEq}/l\right)|\;12000\;(/{\mathrm{mm}}^{3})|\;93(\%)|\;105\;(\mathrm{mmHg})],\;\mathrm{and}\\ T_{4}=[0.51\;\left(\mathrm{mEq}/l\right)|\;6500\;(/{\mathrm{mm}}^{3})|\;99(\%)|\;95\;(\mathrm{mmHg})]. \end{array}$$

By adding the respective units e.g., ‘(mEq/l)’ for each component of the ‘T_j_’s, a modulo addition format for the ‘T_j_’s with the accompanying individual units is expressed as follows:

$$\begin{array}{l} T_{1}=[0.01\left\{0\;\left(\mathrm{mod}\;600\right)\right\}(\mathrm{mEq}/l)|\;4000\;\left(\mathrm{mod}\;50000\right)\left(/{\mathrm{mm}}^{3}\right)|\;98\;\left(\mathrm{mod}\;100\right)\\ \;\times (\%)|\;120\;\left(\mathrm{mod}\;300\right)(\mathrm{mmHg})],\\ T_{2}=[0.01\left\{10\;\left(\mathrm{mod}\;600\right)\right\}(\mathrm{mEq}/l)|\;5400\;\left(\mathrm{mod}\;50000\right)\left(/{\mathrm{mm}}^{3}\right)|\;82\;\left(\mathrm{mod}\;100\right)\\ \;\times (\%)|\;145\;\left(\mathrm{mod}\;300\right)(\mathrm{mmHg})],\\ T_{3}=[0.011\left\{15\;\left(\mathrm{mod}\;600\right)\right\}(\mathrm{mEq}/l)|\;12000\;\left(\mathrm{mod}\;50000\right)\left(/{\mathrm{mm}}^{3}\right)|\;93\;\left(\mathrm{mod}\;100\right)\\ \;\times (\%)|\;105\;\left(\mathrm{mod}\;300\right)(\mathrm{mmHg})],\;\mathrm{and}\\ T_{4}=[0.01\left\{51\;\left(\mathrm{mod}\;600\right)\right\}(\mathrm{mEq}/l)|\;6500\;\left(\mathrm{mod}\;50000\right)\left(/{\mathrm{mm}}^{3}\right)|\;99\;\left(\mathrm{mod}\;100\right)\\ \;\times (\%)|\;95\;\left(\mathrm{mod}\;300\right)(\mathrm{mmHg})] \end{array}$$

**Figure 3:**
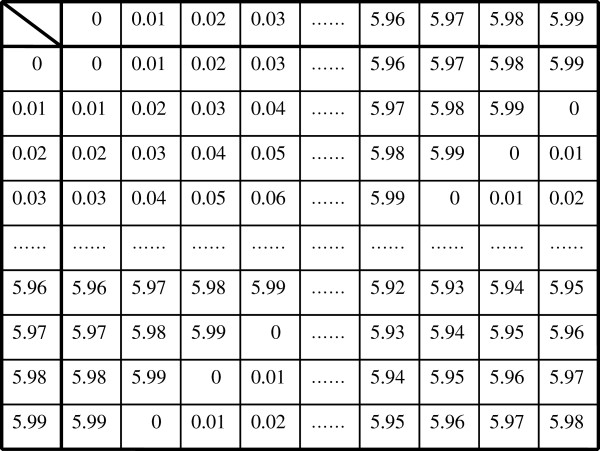
**Cayley table for [Li**^**+**^**]; blood concentration of ‘lithium carbonate’.** Divided by 0.01, the decimal numbers are associated with C_600_={0,1,2,3,…,598,599} satisfying modulo 600 addition.

**Figure 4:**
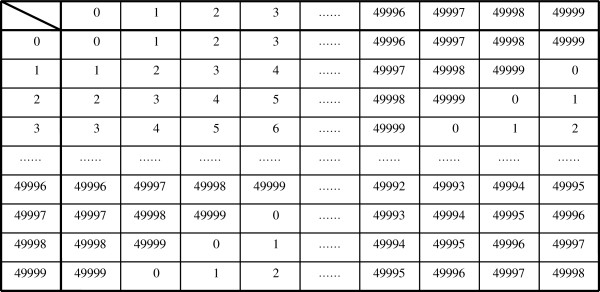
**Cayley table for the modulo group C**_**50000**_**={0,1,2,3,…,49997,49998,49999} with modulo 50000 addition.** Numbers representing the elements of the group are associated with white blood cell (WBC) counts (per mm^3^).

**Figure 5:**
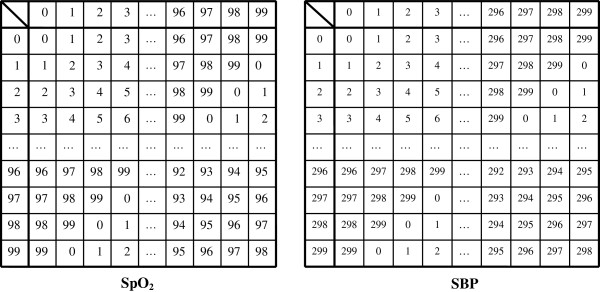
**Cayley tables for percutaneous oxygen saturation (SpO**_**2**_**), and systolic blood pressure (SBP).** In the left table, the integers represent elements of C_100_={0,1,2,3,…,97,98,99} satisfying modulo 100 addition. Similarly, for the right table, the integers are associated with C_300_={0,1,2,3,…,297,298,299} with modulo 300 addition.

Similar to the ‘S_j_’s,‘T_j_’s also obey the group postulates so long as the components of T_j_ reside in the pre-specified ranges. In other words, we have a group T= {T_j_ (j = 1,.., W_T_)| T_j_ ∈ Z_600_×Z_50000_×Z_100_×Z_300_ }, the set of all possible data based on four distinct clinical values. |T| (the order of group T) is |T|≡600×50000×100×300 (Cayley tables are shown in Figures 3, 4, 5). 

In a similar manner for S, the operators ‘ T_(j → k)_ ≡ T_j_^− 1^ * T_k_ = T_k_ − T_j_ ’ can be constructed (see Appendix C for details):

$$\begin{array}{l} T_{\left(1\to 2\right)}=[0.01\left\{10\;\left(\mathrm{mod}\;600\right)\right\}(\mathrm{mEq}/l)|\;1400\;\left(\mathrm{mod}\;50000\right)\left(/{\mathrm{mm}}^{3}\right)|\;84\;\left(\mathrm{mod}\;100\right)\\ \;\times (\%)|\;25\;\left(\mathrm{mod}\;300\right)(\mathrm{mmHg})],\\ T_{\left(2\to 3\right)}=[0.01\left\{5\;\left(\mathrm{mod}\;600\right)\right\}(\mathrm{mEq}/l)|\;6600\;\left(\mathrm{mod}\;50000\right)\left(/{\mathrm{mm}}^{3}\right)|\;11\;\left(\mathrm{mod}\;100\right)\\ \;\times (\%)|\;260\;\left(\mathrm{mod}\;300\right)(\mathrm{mmHg})],\mathrm{and}\\ T_{\left(3\to 4\right)}=[0.01\left\{36\;\left(\mathrm{mod}\;600\right)\right\}(\mathrm{mEq}/l)|\;44500\;\left(\mathrm{mod}\;50000\right)\left(/{\mathrm{mm}}^{3}\right)|\;6\;\left(\mathrm{mod}\;100\right)\\ \;\times (\%)|\;290\;\left(\mathrm{mod}\;300\right)(\mathrm{mmHg})]. \end{array}$$

Also, transitions can naturally be traced iteratively (details given in Appendix D). Thus

$$\begin{array}{ll} T_{1}*T_{\left(1\to 2\right)}*T_{\left(2\to 3\right)}*T_{\left(3\to 4\right)} & ={\;T}_{1}+{\;T}_{\left(1\to 2\right)}+{\;T}_{\left(2\to 3\right)}+{\;T}_{\left(3\to 4\right)}\\ & =[0.01\left\{51\;\left(\mathrm{mod}\;600\right)\right\}(\mathrm{mEq}/l)|\;6500\;\left(\mathrm{mod}\;50000\right)\left(/{\mathrm{mm}}^{3}\right)|\;99\;\left(\mathrm{mod}\;100\right)(\%)|\;95\;\left(\mathrm{mod}\;300\right)(\mathrm{mmHg})].\\ & =[0.51(\mathrm{mEq}/l)|\;6500\left(/{\mathrm{mm}}^{3}\right)|\;99(\%)|\;95(\mathrm{mmHg})]\\ & ={\;T}_{4}. \end{array}$$

These results imply that medication levels and their changes can be composed as a modulo group, e.g., group S in the three-drug prescription (lithium carbonate, mirtazapine and nitrazepam). Moreover, clinical values (e.g., blood concentration of lithium carbonate ([Li^+^]), white blood cell (WBC), percutaneous oxygen saturation (SpO_2_) and systolic blood pressure (SBP)) can also be expressed by a modulo group T.

#### Applications to the Tumor, Node, Metastasis (TNM) classification of malignant tumors

**Figure 6:**
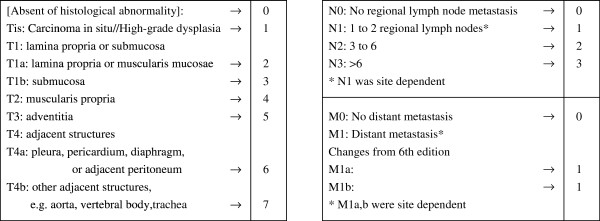
**Example of a modified Esophagus 7th edition, TNM definitions.** A reclassification of malignant tumors of the esophagus is presented. We have added a ‘T0’ entry. Then, the ‘T’ entries are graduated over the range 0–7 (8 grades), ‘N’ over 0–3 (4 grades) and ‘M’ (defined in two ways, for ‘M1a’ and ‘M1b’) over 0–1 (2 grades). According to the number of respective criteria, modulo addition can be introduced and a combination of modular groups, group C = {C_j_ | C_j_ ∈ Z_8_×Z_4_×Z_2_×Z_2_}, signifying that the set of all composited histological disease states of malignant tumors is definable.

The TNM classification of malignant tumors, especially esophageal tumors, [8] forms the basis of another application in line with this method. Esophageal tumors are classified according to the grade of severity: T (primary tumor), N (regional lymph nodes metastasis), and M (distant metastasis). ‘T’ has subgrades from Tis (carcinoma in situ) to T4 (adjacent structures), as in Figure 6; we have added ‘T0: Absent of histological abnormality’. As depicted in Figure 6, we allocate scores from ‘0’ to some maximum to specify a sequential grading (right column). For ‘T’, we assign the integer values [T0: 0,Tis: 1, T1a: 2, T1b: 3, T2: 4, T3: 5, T4a: 6, T4b: 7], and hence modulo 8 addition can be defined. For ‘N’, we assign [N0: 0, N1: 1, N2: 2, N3: 3] with modulo 4 addition, and for ‘M’ defined in two ways as ‘Ma’ and ‘Mb’, for ‘M1a’ and ‘M1b’; [M0a: 0, M1a: 1], [M0b: 0, M1b: 1] with modulo 2 addition (In this regard, ‘Ma’ and ‘Mb’ are complementary, one or other should be ‘0’). Based on these assignments, the following state vectors C_j_ represent simple examples of a patient’s condition:

$$\begin{array}{l} C_{j}=\left[\text{T}\;\left(\mathrm{mod}\;8\right)\left|\text{N}\;\left(\mathrm{mod}\;4\right)\right|\mathrm{Ma}\;\left(\mathrm{mod}\;2\right)|\mathrm{Mb}\;\left(\mathrm{mod}\;2\right)\right],\\ C_{1}=\left[1\;\left(\mathrm{mod}\;8\right)\left|0\;\left(\mathrm{mod}\;4\right)\right|0\;\left(\mathrm{mod}\;2\right)|0\;\left(\mathrm{mod}\;2\right)\right],\\ C_{2}=\left[3\;\left(\mathrm{mod}\;8\right)\left|1\;\left(\mathrm{mod}\;4\right)\right|0\;\left(\mathrm{mod}\;2\right)|0\;\left(\mathrm{mod}\;2\right)\right],\\ C_{3}=\left[5\;\left(\mathrm{mod}\;8\right)\left|2\;\left(\mathrm{mod}\;4\right)\right|0\;\left(\mathrm{mod}\;2\right)|1\;\left(\mathrm{mod}\;2\right)\right],\;\mathrm{and}\\ C_{4}=\left[4\;\left(\mathrm{mod}\;8\right)\left|3\;\left(\mathrm{mod}\;4\right)\right|0\;\left(\mathrm{mod}\;2\right)|1\;\left(\mathrm{mod}\;2\right)\right]. \end{array}$$

From these, transition operators producing changes in condition are defined next (see Appendix E for details):

$$\begin{array}{l} C_{\left(1\to 2\right)}=\left[2\;\left(\mathrm{mod}\;8\right)\left|1\;\left(\mathrm{mod}\;4\right)\right|0\;\left(\mathrm{mod}\;2\right)|0\;\left(\mathrm{mod}\;2\right)\right],\\ C_{\left(2\to 3\right)}=\left[2\;\left(\mathrm{mod}\;8\right)\left|1\;\left(\mathrm{mod}\;4\right)\right|0\;\left(\mathrm{mod}\;2\right)|1\;\left(\mathrm{mod}\;2\right)\right],\;\mathrm{and}\\ C_{\left(3\to 4\right)}=\left[7\;\left(\mathrm{mod}\;8\right)\left|1\;\left(\mathrm{mod}\;4\right)\right|0\;\left(\mathrm{mod}\;2\right)|0\;\left(\mathrm{mod}\;2\right)\right]. \end{array}$$

Also, in a natural manner, a transitioning over the course of sessions can be established iteratively. For example,

$$C_{1}*C_{\left(1\to 2\right)}*C_{\left(2\to 3\right)}*C_{\left(3\to 4\right)}=C_{1}+C_{\left(1\to 2\right)}+C_{\left(2\to 3\right)}+C_{\left(3\to 4\right)}$$

$$\begin{array}{l} =\left[1\;\left(\mathrm{mod}\;8\right)\left|0\;\left(\mathrm{mod}\;4\right)\right|0\;\left(\mathrm{mod}\;2\right)|0\;\left(\mathrm{mod}\;2\right)\right]+\left[2\;\left(\mathrm{mod}\;8\right)\left|1\;\left(\mathrm{mod}\;4\right)\right|0\;\left(\mathrm{mod}\;2\right)|0\;\left(\mathrm{mod}\;2\right)\right]\\ +\left[2\;\left(\mathrm{mod}\;8\right)\left|1\;\left(\mathrm{mod}\;4\right)\right|0\;\left(\mathrm{mod}\;2\right)|1\;\left(\mathrm{mod}\;2\right)\right]+\left[7\;\left(\mathrm{mod}\;8\right)\left|1\;\left(\mathrm{mod}\;4\right)\right|0\;\left(\mathrm{mod}\;2\right)|0\;\left(\mathrm{mod}\;2\right)\right]\\ =\left[1+2+2+7\;\left(\mathrm{mod}\;8\right)|0+1+1+1\;\left(\mathrm{mod}\;4\right)|0+0+0+0\;\left(\mathrm{mod}\;2\right)|0+0+1+0\;\left(\mathrm{mod}\;2\right)\right]\\ =\left[12\;\left(\mathrm{mod}\;8\right)\left|3\;\left(\mathrm{mod}\;4\right)\right|0\;\left(\mathrm{mod}\;2\right)|1\;\left(\mathrm{mod}\;2\right)\right]\\ =\left[4\;\left(\mathrm{mod}\;8\right)\left|3\;\left(\mathrm{mod}\;4\right)\right|0\;\left(\mathrm{mod}\;2\right)|1\;\left(\mathrm{mod}\;2\right)\right]\\ ={\;C}_{4}. \end{array}$$

**Figure 7:**
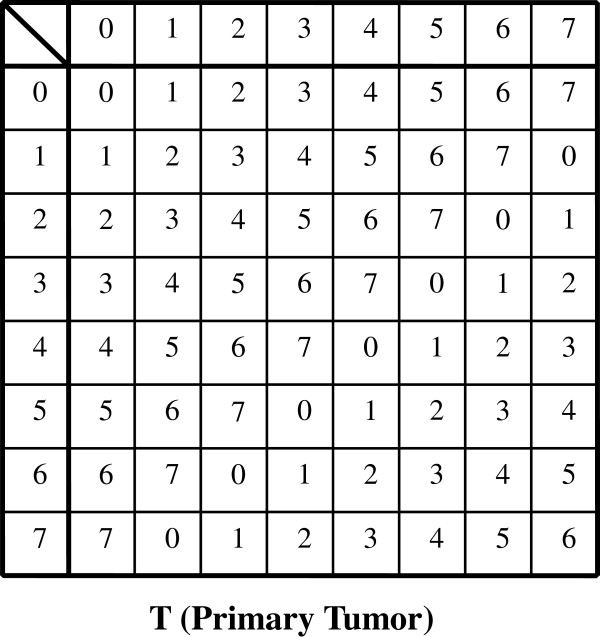
Cayley table for T (primary tumor) graded according to scale 0–7 (8 grades).

**Figure 8:**
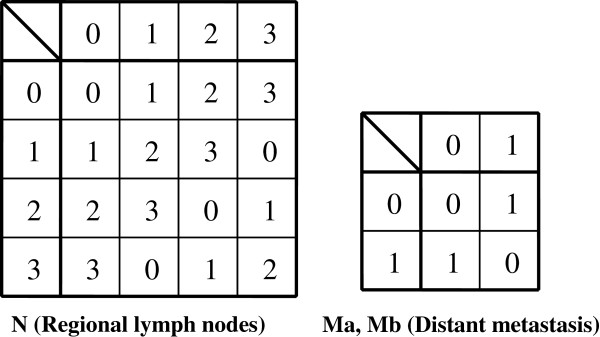
Cayley tables for N (regional lymph nodes) graded by scale 0–3 (4 grades), and M (distant metastasis defined in two ways, for ‘M1a’ and ‘M1b’) graded by scale 0–1 (2 grades).

Hence, these results imply that the TNM classification for malignant tumors can be treated within a single group C. In other words, we can define the group C = {C_j_ (j = 1,…,W_C_)| C_j_ ∈ Z_8_×Z_4_×Z_2_×Z_2_} as the set of all composable disease states in terms of tumor expansion despite containing non-realistic elements such as […|…|1 (mod 2)|1 (mod 2)]. In this regard, |C| (the order of group C) is |C|≡ 8×4×2×2 (Cayley tables are presented in Figures 7 and 8). 

#### Applications to the International Statistical Classification of Diseases and Related Health Problems (ICD)

In the same manner, using the coding schemes of the ICD, almost the same procedures are evident. For instance, a vector-like description containing hypertension (HT), heart failure (HF), diabetes mellitus (DM), panperitonitis, systemic lupus erythematosus (SLE), panic disorder, C-reactive protein (CRP) can be described as follows: for each disease sequential integer scores signify the relative severity with 0 (absence of abnormal sign),…,3 (an average score),…, 6 (extremely severe). Naturally, inclusion of certain aspects of the disease, more detail about the symptomatic domain, and/or laboratory data is possible under the condition that a rigorous ordering of all items can be established that can be indexed from ‘1’, to ‘n’ with n the total number of diseases, symptoms or laboratory data considered. Hence, with

D_j_ = [hypertension (mod 7)|heart failure (mod 7)|diabetes mellitus (mod 7)|panperitonitis (mod 7)|systemic lupus erythematosus (mod 7)|panic disorder (mod 7)| C-reactive protein (mg/l)], examples of possible states would be:

$$\begin{array}{l} D_{1}=[4\;\left(\mathrm{mod}\;7\right)|1\;\left(\mathrm{mod}\;7\right)\left|1\;\left(\mathrm{mod}\;7\right)\right|0\;\left(\mathrm{mod}\;7\right)\left|5\;\left(\mathrm{mod}\;7\right)\right|2\;\left(\mathrm{mod}\;7\right)|4.5\;\left(\mathrm{mg}/l\right)],\\ D_{2}=[3\;\left(\mathrm{mod}\;7\right)|3\;\left(\mathrm{mod}\;7\right)\left|2\;\left(\mathrm{mod}\;7\right)\right|1\;\left(\mathrm{mod}\;7\right)\left|6\;\left(\mathrm{mod}\;7\right)\right|1\;\left(\mathrm{mod}\;7\right)|7.6\;\left(\mathrm{mg}/l\right)],\;\mathrm{and}\\ D_{3}=[4\;\left(\mathrm{mod}\;7\right)|1\;\left(\mathrm{mod}\;7\right)\left|3\;\left(\mathrm{mod}\;7\right)\right|0\;\left(\mathrm{mod}\;7\right)\left|4\;\left(\mathrm{mod}\;7\right)\right|0\;\left(\mathrm{mod}\;7\right)|0.28\;\left(\mathrm{mg}/l\right)]. \end{array}$$

The last component (namely CRP) can be treated in modulo addition by devising an integral expression with an appropriate determination of a modulo divisor so that the maximum CRP would be set at, for example, ‘20 mg/l’ that is expected not to exceed actual laboratory data of CRP. In this instance, the ‘D_j_’s are treatable in the same way as ‘S_j_’s, and ‘T_j_’s, through the definition such as ‘ D_1_ = [… |0.01{450 (mod 2000)} (mg/l)], D_2_ = [… |0.01{760 (mod 2000)} (mg/l)], and D_3_ = [… |0.01{28 (mod 2000)} (mg/l)] ’.

However, in this D_j_ format, a problem arises in that there is an instance of CRP exceeding its assumed maximum value (‘2000’), that is ‘20 mg/l’. With such occurrences, the simplicity of the model might be lost. Therefore, we choose an ordinal sequencing (e.g., ‘0−6’) under the assumption that the grades are defined over intervals; e.g., the CRP scoring ranges might be 0: 0.00−0.02, 1: 0.03−1.00, 2: 1.01−3.00, 3: 3.01−6.00, 4: 6.01−12.0, 5: 12.01−20.00, 6: ≥ 20.01 mg/l and we need to demonstrate that this is in line with procedures. If adopted, D_1_ − D_3_ become:

$$\begin{array}{l} D_{1}=[4\left(\mathrm{mod}\;7\right)|1\;\left(\mathrm{mod}\;7\right)\left|1\;\left(\mathrm{mod}\;7\right)\right|0\;\left(\mathrm{mod}\;7\right)\left|5\;\left(\mathrm{mod}\;7\right)\right|2\;\left(\mathrm{mod}\;7\right)|3\;\left(\mathrm{mod}\;7\right)]\\ \;=\left[4\left|1\right|1\left|0\right|5\left|2\right|3\right]\;\left(\mathrm{mod}\;7\right),\\ D_{2}=[3\left(\mathrm{mod}\;7\right)|3\;\left(\mathrm{mod}\;7\right)\left|2\;\left(\mathrm{mod}\;7\right)\right|1\;\left(\mathrm{mod}\;7\right)\left|6\;\left(\mathrm{mod}\;7\right)\right|1\;\left(\mathrm{mod}\;7\right)|4\;\left(\mathrm{mod}\;7\right)]\\ \;=\left[3\left|3\right|2\left|1\right|6\left|1\right|4\right]\;\left(\mathrm{mod}\;7\right),\;\mathrm{and}\\ D_{3}=[4\left(\mathrm{mod}\;7\right)|1\;\left(\mathrm{mod}\;7\right)\left|3\;\left(\mathrm{mod}\;7\right)\right|0\;\left(\mathrm{mod}\;7\right)\left|4\;\left(\mathrm{mod}\;7\right)\right|0\;\left(\mathrm{mod}\;7\right)|1\;\left(\mathrm{mod}\;7\right)]\;\\ \;=\left[4\left|1\right|3\left|0\right|4\left|0\right|1\right]\;\left(\mathrm{mod}\;7\right). \end{array}$$

Transition operators generating the natural changes in state are defined as follows (see Appendix F for details):

$$\begin{array}{l} D_{\left(1\to 2\right)}=\left[6\left|2\right|1\left|1\right|1\left|6\right|1\right]\;\left(\mathrm{mod}\;7\right)\;\mathrm{and}\\ D_{\left(2\to 3\right)}=\left[1\left|5\right|1\left|6\right|5\left|6\right|4\right]\;\left(\mathrm{mod}\;7\right) \end{array}$$

In the same fashion as ‘C_(j→k)_’s, sequential transitions over the course of sessions can be performed iteratively (A demonstration is presented in Appendix G). Hence

$$D_{1}*D_{\left(1\to 2\right)}*D_{\left(2\to 3\right)}=D_{1}+D_{\left(1\to 2\right)}+D_{\left(2\to 3\right)}$$

$$\begin{array}{l} =\left[4\left|1\right|1\left|0\right|5\left|2\right|3\right]\left(\mathrm{mod}\;7\right)+\left[6\left|2\right|1\left|1\right|1\left|6\right|1\right]\;\left(\mathrm{mod}\;7\right)+\left[1\left|5\right|1\left|6\right|5\left|6\right|4\right]\;\left(\mathrm{mod}\;7\right)\\ =\left[4+6+1\left|1+2+5\right|1+1+1\left|0+1+6\right|5+1+5\left|2+6+6\right|3+1+4\right]\;\left(\mathrm{mod}\;7\right)\\ =\left[11\left|8\right|3\left|7\right|11\left|14\right|8\right]\;\left(\mathrm{mod}\;7\right)\\ =\left[4\left|1\right|3\left|0\right|4\left|0\right|1\right]\left(\mathrm{mod}\;7\right)\\ ={\;D}_{3}. \end{array}$$

**Figure 9:**
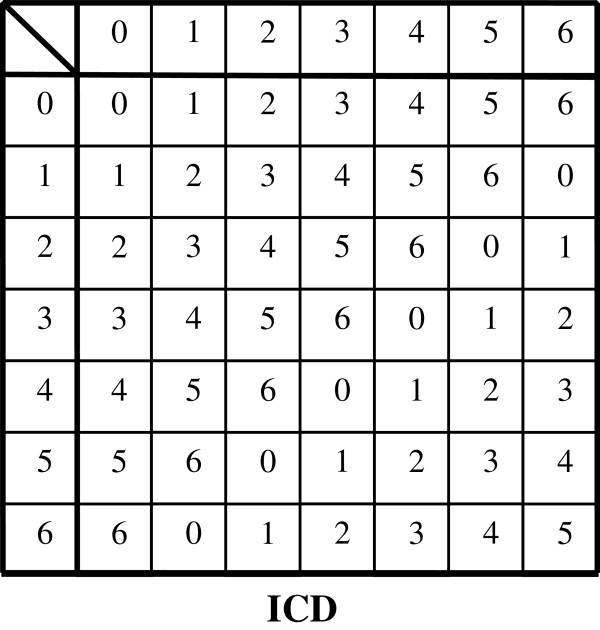
**Cayley table for modulo group C**_**7 **_**with 7 elements for the International Statistical Classification of Diseases and Related Health Problems (ICD).**

Therefore, the ICD coding schemes is also amenable to a modulo group formulation which contain an operator subset that generates all possible transitions regardless of disease severity and laboratory data. Here the group is D = {D_j_ (j = 1,…,W_D_)| D_j_ ∈ Z_7_×Z_7_×Z_7_×…×Z_7_ (n times)} the set of all possible combinations of severities among a number n of diseases and laboratory data. In this regard, |D| (the order of group D) is |D|≡ 7^n^ (A Cayley table is presented in Figure 9). 

## Discussion

In line with the model suggested in our previous article [5], we demonstrate further possible applications using clinical quantities usually seen in daily clinical practices. There, so long as ordinal numbers, including ‘0’ to represent the identity element under modular arithmetic, can reflect the essential characteristics associated with the clinical quantities an appropriately-sized modular group with modulo addition can be constructed. We envisage that if this kind of algorithm is established properly, we can not only monitor patient progress more easily from various perspectives over sessions but also open a door to establish a more refined, sophisticated systematization of clinical medicine, and reduce the futility in the current descriptive style of medicine mentioned in our previous report. The global landscape is ambiguous at the present stage; a more simple and rational landscape might exist by constructing an ideal style based on group-theoretical concepts. However, this is only a presumptive half step but one we believe would be a break-through servicing future medicine. Moreover, we also believe unknown advantages exist in the handling and monitoring of clinical values of patients.

The model proposed is far from complete and there are major restrictions and difficulties in applying it immediately in clinical treatment. Therefore, those limitations of our proposal should be noted here. First, it is doubtful that grading medicine dosage (or various clinical quantities) is appropriate; whether the assumption that dosage levels have the same step-wise increments is suitable to apply in all instances requires assessing. One of the merits of the proposed model is that we can treat clinical values using a single group regardless whether the clinical values are absolute quantities or not (there might be relative differences among various states containing treatment as an operator). This confusion might generate considerable futilities and/or disadvantages in the data. This is a crucially unavoidable issue that needs to be examined thoroughly through rigorous methodology.

Second, the value of the divisor ‘x_i_’ that defines the modulo operation is not always suitable because those values dealt with in this article have not always been optimal. For instance, if the WBC is ‘55000/mm^3^’ under modulo 50000 addition this becomes 5000, which has little significance in our clinical experiences. This problem can be avoided if we select a larger divisor, say x_i_ = 60000. However, coping with the problem in this manner does not provide a resolution. There is a possibility that larger divisors might occur in not only WBC data but also [Li^+^] and SBP (mmHg) as well as clinical examination data. Furthermore, if we avoid this problem by taking sufficiently large divisors, then this yields another type of problem; the modular inverse of certain values can yield large values. For example, under modulo 200000 addition and a WBC value of 12000, the inverse of 12000 is then ‘188000 (= 200000–12000)’. Such values might be too large to be treated in ordinal order when used as a laboratory result. To construct the most convenient model, divisors should be optimized to suitable values that yield more appropriate clinical data. Optionally, indexed descriptions, for instance, might be possible; WBC values of ‘75000’ under modulo 20000 addition are expressed as ‘75000 = 20000×3 + 15000 = 15000_3_ (mod 20000)’, although the index number (viz., subscript ‘_3_’) is only a mark, and ‘15000_3_’ should be treated as merely the element ‘15000’ in modulo group C_20000_. Alternatively, if we decide to focus on the specific range of objects, it might be better to use a common value, such as WBC = 20000, and ignore data values exceeding it; by ignoring large numbers, we can focus instead on the modular set, which in so doing might actually provide advantages in data assessment. Thus, advantages and disadvantages arise together.

Similarly, a large divisor would also be problematic in prescribing medicines such as mirtazapine. Some psychiatrists might medicate mirtazapine over the ordinal-assumed maximum dose, e.g., 45 mg/d. In that instances, we cannot prescribe a mirtazapine dose of 60 mg/d in modulo 4 addition from 15·{0, 1, 2 or 3} mg/d. By raising the maximum dose from 45 to 60 mg/d, we can avoid such issues, noting though that there may be a need for treatments outside the stipulated range. Moreover, transitions associated with ‘highest dosage state back to lowest’ and ‘high-grade tumor to tumor free’ resulting from the maximum grade being changed into the minimum grade’ might become an departure from our clinical experiences. Here, we have only exploited cyclic groups Z and held that the results should be interpreted as if these were a linear measure limited within a finite range (less than the order of Z) even when the results of operations exceed the order of Z. However, medical data, such as those treated in the present article, could be treated not by cyclic Z groups, as for instance ‘S’ and ‘T’, but rather by some structure that may better reflect clinical expectations like groupoids where only the closure law is postulated. From this standpoint, the more suitable aspects that are less likely to produce paradoxes may be those exemplified by ‘C_j_’s and ‘D_j_’s where only a finite grading, ‘0–6’, arises. For these reasons, more effective schemes need to be developed in future studies.

Third, it is always possible to combine any two vectors based on this model; e.g., S_j_ and T_j_ can be combined into a unique vector respecting the definition of each individual component, e.g., U_j_ = [S_j_|T_j_] = [100{3 (mod 13)} (mg/d)| 15{2 (mod 4)} (mg/d)|5{0 (mod 3)} (mg/d)| 0.01{10 (mod 600)} (mEq/l)| 5400 (50000) (/mm^3^)| 82 (mod 100) (*%*)| 145 (mod 300) (mmHg)]. Thus, peculiar combinations of the data sets are likely to occur. For example, the above Cartesian vector U_j_ has a clear mathematical basis but S_j_ and T_j_ might have in combination little direct clinical connections. In other words, large vectors might contain many trivial, similar, redundant and unrelated items which become less meaningful in terms of a clinical standpoint. This is crucially antithetical to the intention of our model and a truly reasonable methodology is necessary to cope with this issue.

Fourth, as stated previously, the model sidesteps the use of negative numbers using instead just natural numbers for graded scoring, e.g., ‘0–6’, or replacing negative quantities with its inverse. Coping with the latter might yield distortions in the data. Basically, the model becomes more useful when it treats: 1) the concentrations of substances that deviate from normal human body levels as ordinal states (for example medicine or specific immuno-antibodies) unless strict limits in their use (such as administration) are determined; and 2) the cases where the grades of severity (not the degree of wellness of body and mind) include the ‘0’ state as an absence of abnormal data/signs (the presence of a normal state), and those that are non-gradable meaning worse states above the pre-determined maximum number (for example ‘6’). The conversion between row data and graded scorings is in general effective with data based on positive numbers. This lack of symmetry in data with respect to positive and negative numbers is a problem that should be resolvable under a rigorous methodology.

Fifth, although in our previous models the ideally healthy state of persons is expressed as an identity element ‘E’, represented by ‘grade 0’, in the present article, ‘0’ is genuinely interpreted as a zero (whatever the unit accompanying it) that produces according to modulo additions no change when acting on other data component. Therefore, in a more expansive sense, ‘0’ may not always signify the most desired state of the laboratory data set (e.g., in the case of T_j_). Of course, among vectors representing disease severity, the ideally desirable (healthy) state is ‘E = [0|0|0|…|0]’; however, this postulate is met in S_j_, as well as C_j_ and D_j_, through strict definition. As for other applications such as medication, blood concentration of medicine (e.g.,[Li^+^]), and WBC, even if the scores are graded in ‘0–6’, ‘grade 0’ does not always represent the healthy state. The regular (standard) ranges of various laboratory quantities usually exist, and these are the truly desirable proportions of clinical values. We should keep in mind such differences in interpretations of ‘0’s.

Sixth, of the various types of modulo operation, only modulo addition has been used in the present article. One of the reasons is the number of grades is not always a prime number, although the modulo operation based on a prime is convenient in modeling [10,11]. Modulo groups based on prime numbers ensure existence of a unique inverse element, without which the possibility of extending the model to ‘rings’ and ‘fields’ is lost. If in the future our models were to be represented in matrix form, this lack of uniqueness might become a considerable disadvantage, for instance, when using inverses of matrices. Although in this model clinical data might not always have a genuine realization by groups, rings, or fields (semi-groups were mentioned in our previous report as a possibility), it can be advantageous to construct a realization that obeys ring or field axioms, even if artificially. Thus, a fundamental resolution of this problem is desired.

Seventh, the present model records and describes each patient’s data set retrospectively. Needless to say, the most valuable information in science is that which is predictive either quantitatively or qualitatively following empirical rules. This model is not considered effective in performing such predictions, at least, in the present form. Perhaps by adding standard deviations ‘±S.D.’ to averages of each data component, trends in scores could then be overviewed given that sufficient data are recorded with the stipulation that each ‘S.D.’ is defined for various types of component combinations at various sessions. For these reasons, future studies are necessary.

Eighth and finally, although strange to say, combining disease states and other clinical quantities as vectors, i.e., independent products of severity levels and/or laboratory quantities, might not always best describe the total disease state. From another perspective, the total disease state is not always composed as a Cartesian vector comprising the respective symptoms and/or individual laboratory data. A total state is not always the product of its partial components, so, the Cartesian vector expression might serve initially to describe and record each clinical quantity independently, but subsequent unexpected connections and/or biases can undermine our simple analysis or obscure links that might exist among individual components. In the kind of model based on linear relationships that we have described, this is a crucial issue to treat.

However, as was mentioned in our previous report, we emphasize that to encapsulate the descriptive style of medicine by group postulates is underscored by the fact that behaviors of certain models can be treated generally and unexceptionally so that genuine mathematical methods can be performed on such models. Therefore, we desire that future studies of such issues can be explored in more effective forms. Examples illustrated in this article hopefully provide further understanding for readers of the model so that for future development more rigorous investigations can be conducted where more generalized operations can effect a significant contribution.

## Conclusions

Within the limited scope of our methodology, there are grounds where other clinical quantities (prescription of medicine, laboratory data, TNM classification of malignant tumors, and ICD coding schemes) can be also treatable with the same group-theory approach as was suggested for psychiatric disease states in our previous report.

## Appendices

In the following appendices A–G, we provide explicit calculations of various operations using the modulo groups S, T, C, and D to be found in the text.

### Appendix A

$$S_{\left(1\to 2\right)}=S_{2}-S_{1}$$

$$\begin{array}{l} =\left[100\left\{2\;\left(\mathrm{mod}\;13\right)\right\}\left|\;15\left\{1\;\left(\mathrm{mod}\;4\right)\right\}\right|\;5\left\{1\;\left(\mathrm{mod}\;3\right)\right\}\right]\;\left(\mathrm{mg}/d\right)\\ -\left[100\left\{0\;\left(\mathrm{mod}\;13\right)\right\}\left|\;15\left\{0\;\left(\mathrm{mod}\;4\right)\right\}\right|\;5\left\{0\;\left(\mathrm{mod}\;3\right)\right\}\right]\;\left(\mathrm{mg}/d\right)\\ =\left[100\left\{2–0\left(\mathrm{mod}\;13\right)\right\}\;\left|15\left\{1–0\left(\mathrm{mod}\;4\right)\right\}\right|\;5\left\{1–0\left(\mathrm{mod}\;3\right)\right\}\right]\;\left(\mathrm{mg}/d\right)\\ =\left[100\left\{2\;\left(\mathrm{mod}\;13\right)\right\}\left|\;15\left\{1\;\left(\mathrm{mod}\;4\right)\right\}\right|\;5\left\{1\;\left(\mathrm{mod}\;3\right)\right\}\right]\;\left(\mathrm{mg}/d\right) \end{array}$$

$$S_{\left(2\to 3\right)}=S_{3}-S_{2}$$

$$\begin{array}{l} =\left[100\left\{4\;\left(\mathrm{mod}\;13\right)\right\}\left|\;15\left\{2\;\left(\mathrm{mod}\;4\right)\right\}\right|\;5\left\{2\;\left(\mathrm{mod}\;3\right)\right\}\right]\;\left(\mathrm{mg}/d\right)\\ -\left[100\left\{2\;\left(\mathrm{mod}\;13\right)\right\}\left|\;15\left\{1\;\left(\mathrm{mod}\;4\right)\right\}\right|\;5\left\{1\;\left(\mathrm{mod}\;3\right)\right\}\right]\;\left(\mathrm{mg}/d\right)\\ =\left[100\left\{4–2\left(\mathrm{mod}\;13\right)\right\}\;\left|15\left\{2–1\left(\mathrm{mod}\;4\right)\right\}\right|\;5\left\{2–1\left(\mathrm{mod}\;3\right)\right\}\right]\;\left(\mathrm{mg}/d\right)\\ =\left[100\left\{2\;\left(\mathrm{mod}\;13\right)\right\}\left|\;15\left\{1\;\left(\mathrm{mod}\;4\right)\right\}\right|\;5\left\{1\;\left(\mathrm{mod}\;3\right)\right\}\right]\;\left(\mathrm{mg}/d\right) \end{array}$$

$$S_{\left(3\to 4\right)}=S_{4}-S_{3}$$

$$\begin{array}{l} =\left[100\left\{6\;\left(\mathrm{mod}\;13\right)\right\}\left|\;15\left\{3\;\left(\mathrm{mod}\;4\right)\right\}\right|\;5\left\{1\;\left(\mathrm{mod}\;3\right)\right\}\right]\;\left(\mathrm{mg}/d\right)\\ -\left[100\left\{4\;\left(\mathrm{mod}\;13\right)\right\}\left|\;15\left\{2\;\left(\mathrm{mod}\;4\right)\right\}\right|\;5\left\{2\;\left(\mathrm{mod}\;3\right)\right\}\right]\;\left(\mathrm{mg}/d\right)\\ =\left[100\left\{6–4\left(\mathrm{mod}\;13\right)\right\}\;\left|15\left\{3–2\left(\mathrm{mod}\;4\right)\right\}\right|\;5\left\{1–2\left(\mathrm{mod}\;3\right)\right\}\right]\;\left(\mathrm{mg}/d\right)\\ =\left[100\left\{2\;\left(\mathrm{mod}\;13\right)\right\}\left|\;15\left\{1\;\left(\mathrm{mod}\;4\right)\right\}\right|\;5\left\{-1\;\left(\mathrm{mod}\;3\right)\right\}\right]\;\left(\mathrm{mg}/d\right)\\ =\left[100\left\{2\;\left(\mathrm{mod}\;13\right)\right\}\left|\;15\left\{1\;\left(\mathrm{mod}\;4\right)\right\}\right|\;5\left\{2\;\left(=-1+3\right)\;\left(\mathrm{mod}\;3\right)\right\}\right]\;\left(\mathrm{mg}/d\right) \end{array}$$

$$S_{\left(4\to 5\right)}=S_{5}-S_{4}$$

$$\begin{array}{l} =\left[100\left\{3\;\left(\mathrm{mod}\;13\right)\right\}\left|\;15\left\{2\;\left(\mathrm{mod}\;4\right)\right\}\right|\;5\left\{0\;\left(\mathrm{mod}\;3\right)\right\}\right]\;\left(\mathrm{mg}/d\right)\\ -\left[100\left\{6\;\left(\mathrm{mod}\;13\right)\right\}\left|\;15\left\{3\;\left(\mathrm{mod}\;4\right)\right\}\right|\;5\left\{1\;\left(\mathrm{mod}\;3\right)\right\}\right]\;\left(\mathrm{mg}/d\right)\\ =\left[100\left\{3–6\left(\mathrm{mod}\;13\right)\right\}\;\left|15\left\{2–3\left(\mathrm{mod}\;4\right)\right\}\right|\;5\left\{0–1\left(\mathrm{mod}\;3\right)\right\}\right]\;\left(\mathrm{mg}/d\right)\\ =\left[100\left\{-3\;\left(\mathrm{mod}\;13\right)\right\}\;\left|15\left\{-1\;\left(\mathrm{mod}\;4\right)\right\}\right|\;5\left\{-1\;\left(\mathrm{mod}\;3\right)\right\}\right]\;\left(\mathrm{mg}/d\right)\\ =\left[100\left\{10\;\left(=-3+13\right)\;\left(\mathrm{mod}\;13\right)\right\}\left|\;15\left\{3\;\left(=-1+4\right)\;\left(\mathrm{mod}\;4\right)\right\}\right|\right)\\ \left(\;5\left\{2\;\left(=-1+3\right)\;\left(\mathrm{mod}\;3\right)\right\}\right]\left(\mathrm{mg}/d\right). \end{array}$$

### Appendix B

$$S_{1}*S_{\left(1\to 2\right)}*S_{\left(2\to 3\right)}*S_{\left(3\to 4\right)}*S_{\left(4\to 5\right)}$$

$$\begin{array}{l} ={\;S}_{1}+{\;S}_{\left(1\to 2\right)}+{\;S}_{\left(2\to 3\right)}+{\;S}_{\left(3\to 4\right)}+{\;S}_{\left(4\to 5\right)}\\ =\left[100\left\{0\;\left(\mathrm{mod}\;13\right)\right\}\left|\;15\left\{0\;\left(\mathrm{mod}\;4\right)\right\}\right|\;5\left\{0\;\left(\mathrm{mod}\;3\right)\right\}\right]\;\left(\mathrm{mg}/d\right)\\ +\left[100\left\{2\;\left(\mathrm{mod}\;13\right)\right\}\left|\;15\left\{1\;\left(\mathrm{mod}\;4\right)\right\}\right|\;5\left\{1\;\left(\mathrm{mod}\;3\right)\right\}\right]\;\left(\mathrm{mg}/d\right)\\ +\left[100\left\{2\;\left(\mathrm{mod}\;13\right)\right\}\left|\;15\left\{1\;\left(\mathrm{mod}\;4\right)\right\}\right|\;5\left\{1\;\left(\mathrm{mod}\;3\right)\right\}\right]\;\left(\mathrm{mg}/d\right)\\ +\left[100\left\{2\;\left(\mathrm{mod}\;13\right)\right\}\left|\;15\left\{1\;\left(\mathrm{mod}\;4\right)\right\}\right|\;5\left\{2\;\left(\mathrm{mod}\;3\right)\right\}\right]\;\left(\mathrm{mg}/d\right)\\ +\left[100\left\{10\;\left(\mathrm{mod}\;13\right)\right\}\left|\;15\left\{3\;\left(\mathrm{mod}\;4\right)\right\}\right|\;5\left\{2\;\left(\mathrm{mod}\;3\right)\right\}\right)\;\left(\mathrm{mg}/d\right)\\ =\left[100\left\{0+2+2+2+10\;\left(\mathrm{mod}\;13\right)\right\}\left|\;15\left\{0+1+1+1+3\;\left(\mathrm{mod}\;4\right)\right\}\right|\right)\\ \left(\;5\left\{0+1+1+2+2\;\left(\mathrm{mod}\;3\right)\right\}\right]\;\left(\mathrm{mg}/d\right)\\ =\left[100\left\{16\;\left(\mathrm{mod}\;13\right)\right\}\left|\;15\left\{6\;\left(\mathrm{mod}\;4\right)\right\}\right|\;5\left\{6\;\left(\mathrm{mod}\;3\right)\right\}\right]\;\left(\mathrm{mg}/d\right)\\ =\left[100\left\{3\;\left(\mathrm{mod}\;13\right)\right\}\left|\;15\left\{2\;\left(\mathrm{mod}\;4\right)\right\}\right|\;5\left\{0\;\left(\mathrm{mod}\;3\right)\right\}\right]\;\left(\mathrm{mg}/d\right)\\ =\left[300\;\left(\mathrm{mg}/d\right)\left|\;30\;\left(\mathrm{mg}/d\right)\right|\;0\;\left(\mathrm{mg}/d\right)\right]. \end{array}$$

### Appendix C

$$T_{\left(1\to 2\right)}=T_{2}-T_{1}$$

$$\begin{array}{l} =[0.01\left\{10\;\left(\mathrm{mod}\;600\right)\right\}\;\left(\mathrm{mEq}/l\right)|\;5400\;\left(\mathrm{mod}\;50000\right)\left(/{\text{mm}}^{3}\right)|\\ \;\;82\;\left(\mathrm{mod}\;100\right)(\%)|\;145\;\left(\mathrm{mod}\;300\right)\;\left(\mathrm{mmHg}\right)]\\ -[0.01\left\{0\;\left(\mathrm{mod}\;600\right)\right\}\;\left(\mathrm{mEq}/l\right)|\;4000\;\left(\mathrm{mod}\;50000\right)\left(/{\mathrm{mm}}^{3}\right)|\\ \;\;98\;\left(\mathrm{mod}\;100\right)(\%)|\;120\;\left(\mathrm{mod}\;300\right)\;\left(\mathrm{mmHg}\right)]\\ =[0.01\{10–0\left(\mathrm{mod}\;600\right)\}\;\left(\mathrm{mEq}/l\right)|5400–4000\left(\mathrm{mod}\;50000\right)\left(/{\mathrm{mm}}^{3}\right)|\\ \;82–98\left(\mathrm{mod}\;100\right)(\%)|145–120\left(\mathrm{mod}\;300\right)\;\left(\mathrm{mmHg}\right)]\\ =[0.01\left\{10\;\left(\mathrm{mod}\;600\right)\right\}\;\left(\mathrm{mEq}/l\right)|\;1400\;\left(\mathrm{mod}\;50000\right)\left(/{\mathrm{mm}}^{3}\right)|\\ \;-16\;\left(\mathrm{mod}\;100\right)(\%)|\;25\;\left(\mathrm{mod}\;300\right)\;\left(\mathrm{mmHg}\right)]\\ =[0.01\left\{10\;\left(\mathrm{mod}\;600\right)\right\}\;\left(\mathrm{mEq}/l\right)|\;1400\;\left(\mathrm{mod}\;50000\right)\left(/{\mathrm{mm}}^{3}\right)|\;84\;\left(=-16+100\right)\\ \;\left(\mathrm{mod}\;100\right)(\%)|\;25\;\left(\mathrm{mod}\;300\right)\;\left(\mathrm{mmHg}\right)] \end{array}$$

$$T_{\left(2\to 3\right)}=T_{3}-T_{2}$$

$$\begin{array}{l} =[0.01\left\{15\;\left(\mathrm{mod}\;600\right)\right\}\;\left(\mathrm{mEq}/l\right)|\;12000\;\left(\mathrm{mod}\;50000\right)\left(/{\mathrm{mm}}^{3}\right)|\\ \;\;93\;\left(\mathrm{mod}\;100\right)(\%)|\;105\;\left(\mathrm{mod}\;300\right)\;\left(\mathrm{mmHg}\right)]\\ -[0.01\left\{10\;\left(\mathrm{mod}\;600\right)\right\}\;\left(\mathrm{mEq}/l\right)|\;5400\;\left(\mathrm{mod}\;50000\right)\left(/{\mathrm{mm}}^{3}\right)|\\ \;\;82\;\left(\mathrm{mod}\;100\right)(\%)|\;145\;\left(\mathrm{mod}\;300\right)\;\left(\mathrm{mmHg}\right)]\\ =[0.01\{15–10\left(\mathrm{mod}\;600\right)\}\;\left(\mathrm{mEq}/l\right)|12000–5400\left(\mathrm{mod}\;50000\right)\\ \;\left(/{\mathrm{mm}}^{3}\right)|93–82\left(\mathrm{mod}\;100\right)(\%)|105–145\left(\mathrm{mod}\;300\right)\;\left(\mathrm{mmHg}\right)]\\ =[0.01\left\{5\;\left(\mathrm{mod}\;600\right)\right\}\;\left(\mathrm{mEq}/l\right)|\;6600\;\left(\mathrm{mod}\;50000\right)\left(/{\mathrm{mm}}^{3}\right)|\;11\;\left(\mathrm{mod}\;100\right)(\%)|\\ \;-40\;\left(\mathrm{mod}\;300\right)\;\left(\mathrm{mmHg}\right)]\\ =[0.01\left\{5\;\left(\mathrm{mod}\;600\right)\right\}\;\left(\mathrm{mEq}/l\right)|\;6600\;\left(\mathrm{mod}\;50000\right)\left(/{\mathrm{mm}}^{3}\right)|\\ \;\;11\;\left(\mathrm{mod}\;100\right)(\%)|\;260\;\left(=-40+300\right)\;\left(\mathrm{mod}\;300\right)\;\left(\mathrm{mmHg}\right)] \end{array}$$

$$T_{\left(3\to 4\right)}=T_{4}-T_{3}$$

$$\begin{array}{l} =[0.01\left\{51\;\left(\mathrm{mod}\;600\right)\right\}\;\left(\mathrm{mEq}/l\right)|\;6500\;\left(\mathrm{mod}\;50000\right)\left(/{\mathrm{mm}}^{3}\right)|\\ \;\;99\;\left(\mathrm{mod}\;100\right)(\%)|\;95\;\left(\mathrm{mod}\;300\right)\;\left(\mathrm{mmHg}\right)]\\ -[0.01\left\{15\;\left(\mathrm{mod}\;600\right)\right\}\\ \;\left(\mathrm{mEq}/l\right)|\;12000\;\left(\mathrm{mod}\;50000\right)\left(/{\mathrm{mm}}^{3}\right)|\;93\;\left(\mathrm{mod}\;100\right)(\%)|\\ \;\;105\;\left(\mathrm{mod}\;300\right)\;\left(\mathrm{mmHg}\right)]\\ =[0.01\{51–15\left(\mathrm{mod}\;600\right)\}\;\left(\mathrm{mEq}/l\right)|6500–12000\left(\mathrm{mod}\;50000\right)\left(/{\mathrm{mm}}^{3}\right)|\\ \;99–93\left(\mathrm{mod}\;100\right)(\%)|95–105\left(\mathrm{mod}\;300\right)\;\left(\mathrm{mmHg}\right)]\\ =[0.01\left\{36\;\left(\mathrm{mod}\;600\right)\right\}\;\left(\mathrm{mEq}/l\right)|-5500\;\left(\mathrm{mod}\;50000\right)\left(/{\mathrm{mm}}^{3}\right)|\;6\;\left(\mathrm{mod}\;100\right)(\%)|\\ \;-10\;\left(\mathrm{mod}\;300\right)\;\left(\mathrm{mmHg}\right)]\\ =[0.01\left\{36\;\left(\mathrm{mod}\;600\right)\right\}\;\left(\mathrm{mEq}/l\right)|\;44500\;\left(=-5500+50000\right)\;\left(\mathrm{mod}\;50000\right)\left(/{\mathrm{mm}}^{3}\right)|\\ \;\;6\;\left(\mathrm{mod}\;100\right)(\%)|\;290\;\left(=-10+300\right)\;\left(\mathrm{mod}\;300\right)\;\left(\mathrm{mmHg}\right)]. \end{array}$$

### Appendix D

$$T_{1}*T_{\left(1\to 2\right)}*T_{\left(2\to 3\right)}*T_{\left(3\to 4\right)}$$

$$\begin{array}{l} ={\;T}_{1}+{\;T}_{\left(1\to 2\right)}+{\;T}_{\left(2\to 3\right)}+{\;T}_{\left(3\to 4\right)}\\ =[0.01\left\{0\;\left(\mathrm{mod}\;600\right)\right\}\;\left(\mathrm{mEq}/l\right)|\;4000\;\left(\mathrm{mod}\;50000\right)\left(/{\mathrm{mm}}^{3}\right)|\\ \;\;98\;\left(\mathrm{mod}\;100\right)(\%)|\;120\;\left(\mathrm{mod}\;300\right)\;\left(\mathrm{mmHg}\right)]\\ +[0.01\left\{10\;\left(\mathrm{mod}\;600\right)\right\}\;\left(\mathrm{mEq}/l\right)|\;1400\;\left(\mathrm{mod}\;50000\right)\left(/{\mathrm{mm}}^{3}\right)|\\ \;\;84\;\left(\mathrm{mod}\;100\right)(\%)|\;25\;\left(\mathrm{mod}\;300\right)\;\left(\mathrm{mmHg}\right)]\\ +[0.01\left\{5\;\left(\mathrm{mod}\;600\right)\right\}\;\left(\mathrm{mEq}/l\right)|\;6600\;\left(\mathrm{mod}\;50000\right)\left(/{\mathrm{mm}}^{3}\right)|\\ \;\;11\;\left(\mathrm{mod}\;100\right)(\%)|\;260\;\left(\mathrm{mod}\;300\right)\;\left(\mathrm{mmHg}\right)]\\ +[0.01\left\{36\;\left(\mathrm{mod}\;600\right)\right\}\;\left(\mathrm{mEq}/l\right)|\;44500\;\left(\mathrm{mod}\;50000\right)\left(/{\mathrm{mm}}^{3}\right)|\\ \;\;6\;\left(\mathrm{mod}\;100\right)(\%)|\;290\;\left(\mathrm{mod}\;300\right)\;\left(\mathrm{mmHg}\right)]\\ =[0.01\left\{0+10+5+36\;\left(\mathrm{mod}\;600\right)\right\}\\ \;\left(\mathrm{mEq}/l\right)|\;4000+\;1400+\;6600+\;44500\;\left(\mathrm{mod}\;50000\right)\left(/{\mathrm{mm}}^{3}\right)|\;98\;\\ \;+\;84\;+\;11\;+\;6\;\left(\mathrm{mod}\;100\right)(\%)|\;120+\;25\;+\;260+\;290\;\left(\mathrm{mod}\;300\right)\;\left(\mathrm{mmHg}\right)]\\ =[0.01\left\{51\;\left(\mathrm{mod}\;600\right)\right\}\;\left(\mathrm{mEq}/l\right)|\;56500\;\left(\mathrm{mod}\;50000\right)\left(/{\mathrm{mm}}^{3}\right)|\\ \;\;199\;\left(\mathrm{mod}\;100\right)(\%)|\;695\;\left(\mathrm{mod}\;300\right)\;\left(\mathrm{mmHg}\right)]\\ =[0.01\left\{51\;\left(\mathrm{mod}\;600\right)\right\}\;\left(\mathrm{mEq}/l\right)|56500–50000\left(\mathrm{mod}\;50000\right)\\ \;\left(/{\mathrm{mm}}^{3}\right)|199–100\left(\mathrm{mod}\;100\right)(\%)|\;695\;-\;300\times 2\;\left(\mathrm{mod}\;300\right)\;\left(\mathrm{mmHg}\right)]\\ =[0.01\left\{51\;\left(\mathrm{mod}\;600\right)\right\}\;\left(\mathrm{mEq}/l\right)|\;6500\;\left(\mathrm{mod}\;50000\right)\left(/{\mathrm{mm}}^{3}\right)|\;99\;\left(\mathrm{mod}\;100\right)\\ \;(\%)|\;95\;\left(\mathrm{mod}\;300\right)\;\left(\mathrm{mmHg}\right)]. \end{array}$$

### Appendix E

$$\begin{array}{l} C_{\left(1\to 2\right)}=C_{2}-C_{1}=\left[3\;\left(\mathrm{mod}\;8\right)\left|1\;\left(\mathrm{mod}\;4\right)\right|0\;\left(\mathrm{mod}\;2\right)\;|0\;\left(\mathrm{mod}\;2\right)\right]\\ \;-\left[1\;\left(\mathrm{mod}\;8\right)\left|0\;\left(\mathrm{mod}\;4\right)\right|0\;\left(\mathrm{mod}\;2\right)\;|0\;\left(\mathrm{mod}\;2\right)\right] \end{array}$$

$$\begin{array}{l} =[3–1\left(\mathrm{mod}\;8\right)\left|1-0\;\left(\mathrm{mod}\;4\right)\right|0-0\;\left(\mathrm{mod}\;2\right)\;|0-0\;\left(\mathrm{mod}\;2\right)]\\ =\left[2\;\left(\mathrm{mod}\;8\right)\left|1\;\left(\mathrm{mod}\;4\right)\right|0\;\left(\mathrm{mod}\;2\right)\;|0\;\left(\mathrm{mod}\right)\right] \end{array}$$

$$\begin{array}{l} C_{\left(2\to 3\right)}=C_{3}-C_{2}=\left[5\;\left(\mathrm{mod}\;8\right)\left|2\;\left(\mathrm{mod}\;4\right)\right|0\;\left(\mathrm{mod}\;2\right)\;|1\;\left(\mathrm{mod}\;2\right)\right]\\ \;-\left[3\;\left(\mathrm{mod}\;8\right)\left|1\;\left(\mathrm{mod}\;4\right)\right|0\;\left(\mathrm{mod}\;2\right)\;|0\;\left(\mathrm{mod}\;2\right)\right] \end{array}$$

$$\begin{array}{l} =[5–3\left(\mathrm{mod}\;8\right)\left|2-1\;\left(\mathrm{mod}\;4\right)\right|0-0\;\left(\mathrm{mod}\;2\right)\;|1\;-0\;\left(\mathrm{mod}\;2\right)]\\ =\left[2\;\left(\mathrm{mod}\;8\right)\left|1\;\left(\mathrm{mod}\;4\right)\right|0\;\left(\mathrm{mod}\;2\right)\;|1\;\left(\mathrm{mod}\right)\right] \end{array}$$

$$\begin{array}{l} C_{\left(3\to 4\right)}=C_{4}-C_{3}=\left[4\;\left(\mathrm{mod}\;8\right)\left|3\;\left(\mathrm{mod}\;4\right)\right|0\;\left(\mathrm{mod}\;2\right)\;|1\;\left(\mathrm{mod}\;2\right)\right]\\ \;-\left[5\;\left(\mathrm{mod}\;8\right)\left|2\;\left(\mathrm{mod}\;4\right)\right|0\;\left(\mathrm{mod}\;2\right)|1\;\left(\mathrm{mod}\;2\right)\right] \end{array}$$

$$\begin{array}{l} =[4–5\left(\mathrm{mod}\;8\right)\left|3-2\;\left(\mathrm{mod}\;4\right)\right|0-0\;\left(\mathrm{mod}\;2\right)|1\;-\;1\;\left(\mathrm{mod}\;2\right)]\\ =[-1\;\left(\mathrm{mod}\;8\right)\left|1\;\left(\mathrm{mod}\;4\right)\right|0\;\left(\mathrm{mod}\;2\right)|0\;\left(\mathrm{mod}\;2\right)]\\ =\left[7\;\left(\mathrm{mod}\;8\right)\left|1\;\left(\mathrm{mod}\;4\right)\right|0\;\left(\mathrm{mod}\;2\right)|0\;\left(\mathrm{mod}\;2\right)\right]. \end{array}$$

### Appendix F

$$D_{\left(1\to 2\right)}=D_{2}-D_{1}$$

$$\begin{array}{l} =\left[3-4\left|3-1\right|2-1\left|1-0\right|6-5\left|1-2\right|4-3\right]\;\left(\mathrm{mod}\;7\right)\\ =[-1\left|2\right|1\left|1\right|1\left|-1\right|1]\;\left(\mathrm{mod}\;7\right)\\ =\left[6\left|2\right|1\left|1\right|1\left|6\right|1\right]\;\left(\mathrm{mod}\;7\right) \end{array}$$

$$D_{\left(2\to 3\right)}=D_{3}-D_{2}$$

$$\begin{array}{l} =\left[4-3\left|1-3\right|3-2\left|0-1\right|4-6\left|0-1\right|1-4\right]\;\left(\mathrm{mod}\;7\right)\\ =\left[1\left|-2\right|1\left|-1\right|-2\left|-1\right|-3\right]\;\left(\mathrm{mod}\;7\right)\\ =\left[1\left|5\right|1\left|6\right|5\left|6\right|4\right]\;\left(\mathrm{mod}\;7\right). \end{array}$$

### Appendix G

$$D_{1}*D_{\left(1\to 2\right)}*D_{\left(2\to 3\right)}$$

$$\begin{array}{l} ={\;D}_{1}+{\;D}_{\left(1\to 2\right)}+{\;D}_{\left(2\to 3\right)}\\ =\left[4\left|1\right|1\left|0\right|5\left|2\right|3\right]\;\left(\mathrm{mod}\;7\right)\\ +\left[6\left|2\right|1\left|1\right|1\left|6\right|1\right]\;\left(\mathrm{mod}\;7\right)\\ +\left[1\left|5\right|1\left|6\right|5\left|6\right|4\right]\;\left(\mathrm{mod}\;7\right)\\ =\left[4+6+1\left|1+2+5\right|1+1+1\left|0+1+6\right|5+1+5\left|2+6+6\right|3+1+4\right]\;\left(\mathrm{mod}\;7\right)\\ =\left[11\left|8\right|3\left|7\right|11\left|14\right|8\right]\;\left(\mathrm{mod}\;7\right)\\ =\left[4\left|1\right|3\left|0\right|4\left|0\right|1\right]\;\left(\mathrm{mod}\;7\right). \end{array}$$

## Competing interests

The authors declare that they have no competing interests.

## Authors’ contributions

JS conceived of the main idea of this article and wrote the manuscript. SM revised the manuscript. JI gave advice on the potential applicability of the model to clinical research and treatment. All authors read and approved the final manuscript.
